# 3 L split-dose polyethylene glycol is superior to 2 L polyethylene glycol in colonoscopic bowel preparation in relatively high-BMI (≥ 24 kg/m^2^) individuals: a multicenter randomized controlled trial

**DOI:** 10.1186/s12876-023-03068-9

**Published:** 2023-12-05

**Authors:** Hailin Yan, Hongyu Huang, Dailan Yang, Zonghua Chen, Chao Liu, Zhong Huang, Rui Zhao, Jing Shan, Li Yang, Jinlin Yang, Kai Deng

**Affiliations:** 1 Department of Gastroenterology & Hepatology, West China Hospital, Sichuan University, Chengdu, 610041 Sichuan China; 2 Sichuan University-University of Oxford Huaxi Joint Centre for Gastrointestinal Cancer, Chengdu, 610041 Sichuan China; 3 Department of Gastroenterology, West China School of Public Health and West China Fourth Hospital, Sichuan University, Chengdu, 610041 Sichuan China; 4 Department of Gastroenterology, Yibin Second People’s Hospital, Yibin, 644000 Sichuan China; 5 Department of Gastroenterology, Hospital of the Office of the Tibet Autonomous Region People’s Government in Chengdu, Chengdu, 610041 Sichuan China; 6 Division of Gastroenterology, Zigong First People’s Hospital, Zigong, 643000 Sichuan China; 7 Department of Endoscopy Center, Sichuan Cancer Hospital & Institute, Sichuan Cancer Center, Chengdu, 610041 Sichuan China; 8 Department of Gastroenterology, The 3rd People’s Hospital of Chengdu, Southwest Jiaotong University, Chengdu, 610031 Sichuan China

**Keywords:** Body mass index, Bowel preparation, Polyethylene glycol, Polyp detection, Colonoscopy

## Abstract

**Background:**

Whether body mass index (BMI) is a risk factor for poor bowel preparation is controversial, and the optimal bowel preparation regimen for people with a high BMI is unclear.

**Methods:**

We prospectively included 710 individuals with high BMIs (≥ 24 kg/m^2^) who were scheduled to undergo colonoscopy from January to November 2021 at 7 hospitals. Participants were randomly allocated into 3 L split-dose polyethylene glycol (PEG) group (*n*=353) and 2 L PEG group (*n*=357). The primary outcome was the rate of adequate bowel preparation, and the secondary outcomes included Boston Bowel Preparation Scale (BBPS) score, polyp detection rate, cecal intubation rate, and adverse reactions during bowel preparation. Furthermore, we did exploratory subgroup analyses for adequate bowel preparation.

**Results:**

After enrollment, 15 individuals didn’t undergo colonoscopy, finally 345 participants took 3 L split-dose PEG regimen, and 350 participants took 2 L PEG regimen for colonoscopic bowel preparation. 3 L split-dose PEG regimen was superior to 2 L PEG regimen in the rate of adequate bowel preparation (81.2% vs. 74.9%, *P* = 0.045), BBPS score (6.71±1.15 vs. 6.37±1.31, *P* < 0.001), and the rate of polyp detection (62.0% vs. 52.9%, *P* = 0.015). The cecal intubation rate was similar in both groups (99.7%). Regarding adverse reactions, individuals were more likely to feel nausea in the 3 L PEG group (30.9% vs. 19.3%; *P* = 0.001); however, the degree was mild. In the subgroup analysis for adequate bowel preparation, 3 L split-dose PEG regimen performed better than 2 L PEG regimen in the overweight (BMI 25-29.9 kg/m^2^ ) (*P* = 0.006) and individuals with constipation (*P* = 0.044), while no significant differences were observed in relatively normal (BMI 24-24.9 kg/m^2^) (*P* = 0.593) and obese individuals (BMI ≥ 30 kg/m^2^) (*P* = 0.715).

**Conclusions:**

3 L split-dose PEG regimen is superior to 2 L PEG regimen for colonoscopic Bowel Preparation in relatively high-BMI individuals, especially overweight individuals (BMI 25-29.9 kg/m^2^ ).

**Trial Registration:**

This trial was registered in the Chinese Clinical Trials Registry (ChiCTR2000039068). The date of first registration, 15/10/2020, http://www.chictr.org.cn

**Supplementary Information:**

The online version contains supplementary material available at 10.1186/s12876-023-03068-9.

## Introduction

Colorectal cancer (CRC) is the third most common malignancy and the second leading cause of cancer-related mortality worldwide [1]. Multiple risk factors have been reported to be associated with CRC, including obesity [2]. For each 5 kg/m^2^ increase in body mass index (BMI), the risk of CRC will increase by approximately 18% [3]. As the prevalence of overweight and obesity grows, human health will face tremendous challenges. Colonoscopy is important for screening, diagnosing, and treating colorectal lesions, and the success of a colonoscopy is highly dependent on the quality of bowel preparation (BP). Inadequate BP may lead to missed lesions, repeat examinations, increased cost, and even serious complications.

Several risk factors have been reported to be associated with inadequate bowel cleaning, such as age, male sex, constipation, diabetes mellitus, hypertension, cirrhosis, and stroke [4]. However, there have been inconsistent results regarding body mass index (BMI). Retrospective studies reported that obesity was an independent predictor of inadequate BP at colonoscopy [5, 6], while a prospective observational study of 1314 patients revealed that increased BMI was not predictive of suboptimal BP for colonoscopy [7]. Two meta-analyses including 67 and 24 studies, respectively, found inconsistent results regarding BMI and history of colon preparation failure [4, 8].

Polyethylene glycol (PEG) solution is currently the most widely used for bowel cleansing before colonoscopy, and in most west countries, 4 L PEG solution is the standard bowel preparation regimen [9]. However, compared to Westerners, Asians usually have smaller body size, lower body weight, and different diet habits, the large volume of 4 L PEG might be poorly tolerated by the Chinese population [10]. Therefore, it is not recommended to routinely use the 4 L PEG solution for intestinal preparation in our country [11]. In a previous randomized controlled trial, the same-day single dose of 2 L PEG was not inferior to 4 L split-dose PEG in low-risk patients on adequate BP [12]. And the single dose of low-volume regimen had significantly fewer adverse events. However, in that study, researchers excluded patients with BMI >25. Besides, in one prospective study, the researchers found increased BMI was not correlated with suboptimal bowel preparation for colonoscopy when most patients received a split dose 4 L PEG solution [7]. Due to the smaller size of the Chinese population, we chose to compare a 3 L PEG regimen with a 2 L PEG regimen.

Latest guidelines on bowel preparation do not provide recommendations on the appropriate dose of PEG for overweight and obese population [13]. There are now lack of prospective randomized study to answer this question. Therefore, we performed this multicenter, randomized controlled trial (RCT) to explore the optimal method for bowel cleaning using PEG in relatively high-BMI individuals.

## Methods

### Setting and ethics

This study was a multicenter, endoscopist single-blinded RCT. This study was approved by the research ethics boards from all participating hospitals. In addition, we registered at the Chinese Clinical Trial Registry (ChiCTR2000039068).

### Study populations

Patients scheduled to undergo colonoscopy were selected from 7 tertiary hospitals in Sichuan Province from January to November 2021. The inclusion criteria were as follows: (1) patients who underwent colonoscopy for the first time within one month; (2) age of 18-65 years; (3) BMI ≥ 24 kg/m^2^; and (4) signed informed consent. Patients were excluded if any of the following conditions were satisfied: (1) pregnant or lactating women; (2) history of colon resection; (3) serious heart disease (acute heart failure and acute coronary syndrome, serious liver disease (acute liver failure and decompensate cirrhosis belonged to Child-Pugh class C, serious kidney disease (eGFR<15 ml/min), serious lung disease (respiratory failure with PaO2 less than 60 mmHg or PaCO2 higher than 50mmHg). (4) severe electrolyte disorders; (5) gastrointestinal bleeding with hemoglobin level less than 70 g/L; (6) intestinal obstruction, toxic megacolon, severe inflammatory bowel disease; or (7) refusal to use PEG.

### Bowel preparation and colonoscopy

Eligible participants were randomly assigned to the experimental group or the control group. The experimental group was given a 3 L split-dose regimen (1 L PEG was taken at 8:00 pm one day before the colonoscopy date, and 2 L PEG was taken 4-6 h before the colonoscopy). The control group was given a single dose regimen of 2 L PEG, taken 4-6 h before the colonoscopy.

The laxative was PEG electrolyte powder (specification: 68.56 g/bag or 137.15 g/bag, Shenzhen Wanhe Pharmaceutical Co., LTD.), whose main component was PEG 4000. Once enrolled, each participant received a uniform education both verbally and in writing. Participants were guided to consume a low-residue diet one day before the colonoscopy (Supplementary Table 1). During bowel preparation, the participants were instructed to take approximately 250 mL PEG every 15 minutes. Colonoscopy was performed by experienced endoscopists (defined as performing >1000 colonoscopies). In addition, all endoscopists were blinded to the randomization status. Both Narrow band Imaging Endoscopy (NBI) and white light endoscope were used to detect polyps. In our study, the colonoscopy withdrawal time was no less than 6 minutes. For those cases with poor bowel preparation, the withdrawal time could be up to 10 minutes.

### Definition

The bowel preparation quality was assessed using the Boston Bowel Preparation Scale (BBPS) score [14], which was a 4-point scoring system used to assess 3 segments of the colon: the right colon, the transverse colon and the left colon. Score 0: Unprepared colonic segment with mucosa not seen because of solid stool that cannot be cleared. Score 1: Portion of mucosa of the colonic segment seen, but other areas of the colonic segment not well seen because of staining, residual stool, and/or opaque liquid. Score 2: Minor amount of residual staining, small fragments of stool, and/or opaque liquid, but mucosa of colonic segment seen well. Score 3: Entire mucosa of colonic segment seen well, with no residual staining, small fragments of stool, or opaque liquid. The BBPS score was evaluated independently by two endoscopists, and disagreements were resolved by discussion until a consensus was reached. Adequate bowel preparation was defined as a score of ≥2 on all colon segments. Bowel cleanliness was divided into excellent (total score: 8-9), good (total score: 6-7, each segment ≥ 2), fair (total score: 3-5, or total score: 6-7 but any segmental score < 2), and poor (total: score 0-2). The examples of original figures on the cleansing level of colonoscopy were presented in the supplementary Fig. 1.

### Outcome measures

The primary outcome was the rate of adequate bowel preparation (RABP). The secondary outcomes included BBPS score; polyp detection rate (PDR); cecal intubation rate (CIR); and adverse reactions during bowel preparation, including dizziness, weakness, nausea, vomiting, abdominal pain, abdominal distension, and anal pendant expansion. Reports of adverse events were collected by telephone within two weeks after colonoscopy.

### Sample size and randomization method

Based on current research, the RABP in normal-weight people is approximately 80%, while that in people with high BMIs is less than 70%. Therefore, we assumed that the RABP for high-BMI individuals using 3 L PEG would be approximately 80%. The α value was set at 0.05, the β value was set at 0.2, and the lost to follow-up rate was set at 0.2. The sample size calculated by the professional sample size calculation tool (Medsci App 5.6.4) was 696 cases.

Participants from each center were randomly and equally assigned to the 2 L and 3 L groups according to the block randomization schedule, with a block size of 4. The random number tables were independently generated by the China Evidence-based Medicine Center, West China Hospital of Sichuan University, using SAS 9.4 as the generation tool.

### Statistical analysis

IBM SPSS 26.0 was used for statistical analysis. Qualitative data were expressed as frequencies (percentages) and were compared using Person’s χ2 test or Fisher’s exact test, as appropriate. Quantitative data were reported as the mean with standard deviation (SD), or median with interquartile range (IQR). Normally distributed quantitative data were analyzed with a T test, while nonnormally distributed quantitative data were compared using the Mann–Whitney U test. A value of P<0.05 was used for all statistical analyses. Participants were divided into subgroups based on BMI classification s[15] and comorbidities. Moreover, a logistic regression model was used to evaluate risk factors for inadequate bowel preparation.

## Results

**Fig. 1:**
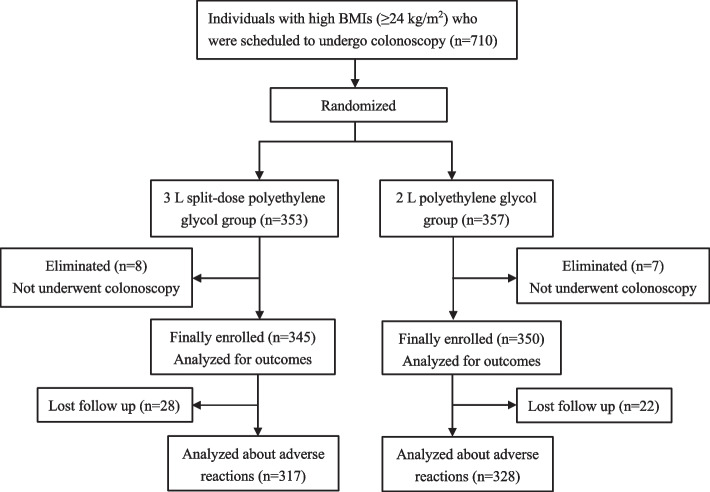
Study flowchart

A total of 710 patients were enrolled from January to November 2021, including 353 individuals in 3 L split dose PEG group and 357 individuals in 2 L PEG group. After enrollment, 8 and 7 individuals in the 3 L and 2 L group, respectively did not undergo colonoscopy at last. Moreover, 28 individuals in 3 L split dose PEG group, and 22 individuals in 2 L PEG group were lost to follow-up, which means we failed to ring them up. Thus, there was a lack of information about adverse reactions for those people (Fig. 1).

### Baseline characteristics

As shown in Table 1, males accounted for 69.0% in 3 L split-dose group and 72.9% in 2 L group, respectively. The average height and weight were 166.4 ± 8.1 cm and 74.4 ± 10.13 kg, respectively, and the BMI was mainly concentrated in the 25-29.9 kg/m^2^ range. Regarding education levels, most of the patients had a high school education (226, 32.5%) or an undergraduate diploma (351, 50.5%). In addition, 164 (23.6%) and 130 (18.7%) patients had a history of smoking or drinking, respectively. The main comorbidities were hypertension and constipation, accounting for 127 (18.3%) and 117 cases (16.8%) among the total individuals. A total of eight patients with compensatory cirrhosis were included, with a total Child-Pugh score of 5, belonged to class A. As for operations, 16 people in the 3 L group (4 appendectomies, 3 cholecystectomies, 9 gynecological operations), and 14 people in the 2 L group (2 appendectomies, 7 cholecystectomies, 5 gynecological operations), respectively, had the history of abdominal operation. No significant difference was found in all comparison between 3L and 2L groups.

**Table 1:** Baseline characteristics of patients

	3 L split-dose group (*n* = 345)	2 L group (*n* = 350)
Male sex, n (%)	238 (69.0)	255 (72.9)
Age, mean ± SD, years	48.7 ± 9.8	48.4 ± 10.5
Height, mean ± SD, cm	165.9 ± 7.8	166.8 ± 8.4
Weight, mean ± SD, kg	73.6 ± 9.5	75.3 ± 10.7
BMI, n (%)		
24-24.9 kg/m^2^	91 (26.4)	74 (21.1)
25-29.9 kg/m^2^	228 (66.1)	241 (68.9)
≥30 kg/m^2^	26 (7.5)	35 (10.0)
Education degree, n (%)		
Primary school	37 (10.7)	37 (10.6)
High school	108 (31.3)	118 (33.7)
Undergraduate degree	175 (50.7)	176 (50.3)
Master's degree	25 (7.2)	29 (5.4)
Smoking, n (%)	71 (20.6)	93 (26.6)
Drinking, n (%)	67 (19.4)	63 (18.0)
Comorbidities, n (%)		
Hypertension	57 (16.5)	70 (20.0)
Diabetes	21 (6.1)	23 (6.6)
Cirrhosis^a^	1 (0.3)	7 (2.0)
Constipation	50 (14.5)	67 (19.1)
History of abdominal surgery, n (%)	16 (4.6)	14 (4.0)
Appendectomy	4 (1.2)	2 (0.6)
Cholecystectomy	3 (0.9)	7 (2.0)
Gynecological operation	9 (2.6)	5 (1.4)

No sigificant difference was found in all comparison between 3L and 2L groups.

^a^All belonged to Child-Pugh class A, with a score of 5.

### Outcomes of bowel preparation

3 L split-dose PEG group achieved a higher RABP in total colon than 2 L PEG group (81.2% vs. 74.9%; *P* = 0.045). For BBPS score, individuals in 3 L PEG group got a higher score than those in 2 L group at the left colon2.33 ± 0.62 vs. 2.18 ± 0.64 (*P* = 0.003), transverse, right, and total colon in the 3 L versus 2 L group were, 2.41 ± 0.55 vs. 2.28 ± 0.58 (*P* = 0.002), 1.98 ± 0.51 vs. 1.91 ± 0.56 (*P* = 0.070) and 6.71 ± 1.15 vs. 6.37 ± 1.31 (*P* < 0.001), respectively. In addition, there was a significant difference in the composition of bowel-cleansing grades between the two groups (*P* = 0.006), and patients using 3 L spilt-dose regimen were more likely to obtain excellent bowel preparation.

Polyps were detected in 214 patients in 3 L split-dose group with a total number of 735 and 183 patients in 2 L PEG group, with a total of 696 polyps. The median number of polyps detected in the 3 L group was 1 (0-3) and that in the 2 L group was 1 (0-2); the difference was statistically significant (*P* = 0.030). The PDR of the 3 L group was higher than that of the 2 L group: 62.0% and 52.9% (*P* = 0.015). The CIRs were nearly 99.7% in both groups. As for other endoscopic findings, we found submucosal tumor in 6 patients of the 3 L PEG group, and in 5 patients of the 2 L PEG group. Besides, colon diverticulum was detected in 17 and 26 patients in the 3 L and 2 L PEG group, respectively (Table 2).

**Table 2:** Outcome of bowel preparation

	3 L split-dose group (*n* = 345)	2 L group (*n* = 350)	*P* value*
Adequate bowel preparation, n (%)			
Left colon	319 (92.5)	313 (89.4)	0.163
Transverse colon	335 (97.1)	330 (94.3)	0.068
Right colon	298 (86.4)	285 (81.4)	0.076
Total	280 (81.2)	262 (74.9)	0.045
BBPS score, mean ± SD			
Left colon	2.33±0.62	2.18±0.64	0.003
Transverse colon	2.41±0.55	2.28±0.58	0.002
Right colon	1.98±0.51	1.91±0.56	0.070
Total	6.71±1.15	6.37±1.31	<0.001
Bowel-cleansing grades, n (%)			0.006
Excellent	91 (26.4)	57 (16.3)	
Good	189 (54.8)	205 (58.6)	
Fair	64 (18.6)	85 (24.3)	
Poor	1 (0.3)	3 (0.9)	
Total polyps detected	735	696	
Polyps per colonoscopy, median (IQR)	1 (0-3)	1 (0-2)	0.030
Polyp detection rate, n (%)	214 (62.0)	183 (52.9)	0.015
Cecal intubation rate, n (%)	344 (99.7)	349 (99.7)	0.992
Other endoscopic findings			
Submucosal tumor	6 (1.7)	5 (1.4)	0.734
Colonic diverticula	17 (4.9)	26 (7.4)	0.171

*P-value was calculated using the χ2-test or Fisher’s exact test for categorical data. *P*-value was calculated using the t-test or Mann– Whitney U-test for continuous data

*BBPS* Boston Bowel Preparation Scale; *IQR* Interquartile Range

### Adverse reactions

28 individuals in the 3 L group and 22 in the 2 L group were lost to follow up, without data on adverse reactions. No serious adverse events requiring medical intervention occurred in either group. As shown in Table 3, participants in the 3 L PEG group were more likely to feel nausea than those in the 2 L PEG group (30.8% vs. 19.3%; *P* = 0.001), but most of the cases were mild. The two groups were comparable regarding the incidence of other adverse events, including dizziness (8.5% vs. 9.0%, *P* = 0.876), vomiting (14.8% vs. 12.1%, *P* = 0.330), abdominal pain (16.4% vs. 11.5%, *P* = 0.078), abdominal distension (30.5% vs. 24.0%, *P* = 0.064), weakness (11.6% vs. 11.5%, *P* = 0.966), and anal pendant expansion (16.8% vs. 12.9%, *P* = 0.147).

**Table 3:** Adverse events of bowel preparation

	3 L split-dose group (*n* = 318)	2 L group (*n* = 321)	*P* value*
Dizzy, n (%)	27 (8.5)	29 (9.0)	0.876
Mild	27 (100)	27 (93.1)	
Moderate	0	2 (6.9)	
Severe	0	0	
Nausea, n (%)	98 (30.8)	62 (19.3)	0.001
Mild	78 (79.6)	56 (90.3)	
Moderate	16 (16.3)	6 (9.7)	
Severe	4 (4.1)	0	
Vomiting, n (%)	47 (14.8)	39 (12.1)	0.330
Mild	38 (80.9)	33 (84.6)	
Moderate	6 (12.8)	5 (12.8)	
Severe	3 (6.4)	1(2.6)	
Abdominal pain, n (%)	52 (16.4)	37 (11.5)	0.078
Mild	48 (92.3)	33 (89.2)	
Moderate	2 (3.8)	4 (10.8)	
Severe	2 (3.8)	0	
Abdominal distension, n (%)	97(30.5)	77 (24.0)	0.064
Mild	76(78.4)	64 (83.1)	
Moderate	19(19.6)	12 (15.6)	
Severe	2(2.1)	1 (1.3)	
Weak, n (%)	37(11.6)	37 (11.5)	0.966
Mild	34(91.9)	34 (91.9)	
Moderate	2(5.4)	2 (5.4)	
Severe	1(2.7)	1(2.7)	
Anal pendant expansion, n (%)	58(16.8)	45(12.9)	0.147
Mild	50(86.2)	41(91.1)	
Moderate	6(10.3)	4(8.9)	
Severe	2(3.4)	0	

*P-value was calculated using the χ2-test or Fisher’s exact test for categorical data

### Subgroup Analysis for adequate bowel prep

Subgroup analysis based on BMI classifications by WHO criteria showed that overweight individuals (BMI 25-29.9 kg/m^2^) in the 3 L split-dose group had a higher RABP than those in the 2 L groups (82.9% vs. 72.2%, *P* = 0.006). However, in relatively normal (BMI 24-24.9 kg/m^2^) and obese individuals (BMI ≥ 30 kg/m^2^), the RABP was similar between the two regimens (Table 4). For individuals with constipation, the 3 L split-dose regimen was superior to the 2 L regimen in ARBP (*P* = 0.044). No significant differences were observed for subgroups based on hypertension (*P* = 0.704) and diabetes (*P* = 0.064).

**Table 4:** Exploratory subgroup analyses for adequate bowel preparation

	3 L split-dose group (*n*=345)			2 L group (*n* = 350)			*P* value*
	All	Adequate BP	RABP(%)	All	Adequate BP	RABP (%)	
BMI classification							
24-24.9 kg/m2	91	72	79.1	74	61	82.4	0.593
25-29.9 kg/m2	228	189	82.9	241	174	72.2	0.006
≥30 kg/m2	26	19	73.1	35	27	77.1	0.715
Comorbidities							
Hypertension	57	44	77.2	52	18	74.3	0.704
Diabetes	21	12	57.1	23	19	82.6	0.064
Cirrhosis	1	1	100	7	4	57.1	-
Constipation	50	43	86.0	67	47	70.1	0.044

*P-value was calculated using the χ2-test or Fisher’s exact test for categorical data

## Discussion

This multicenter randomized controlled trial study confirmed that 3 L split-dose PEG regimen was superior to 2 L PEG regimen in colonoscopic bowel preparation in relatively high-BMI individuals (BMI ≥ 24 kg/m^2^). Furthermore, in the subgroup of overweight individuals (BMI 25-29.9 kg/m^2^) and those with constipation, the advantage was more obvious.

The optimum dose of PEG for bowel preparation before colonoscopy remains a matter of debate. A meta-analysis [16] reported that 4 L split-dose PEG regimen was better than other bowel preparation methods for colonoscopy, with a high odds ratio (OR, 3.46; 95% CI, 2.45-4.89; *P* < 0.01) for excellent or good bowel preparation quality. However, there were significant heterogeneity among studies due to differences in patient demographics and protocols. Besides, several studies recently showed 2 L or 3 L PEG regimen was not inferior to 4 L PEG regimen [17–19]. Unfortunately, all the studies failed to give attention to special groups (e.g., individuals with a high BMI). Due to the smaller size of the Chinese population, we chose to compare a 3 L PEG regimen with a 2 L PEG regimen.

Adequate bowel prep is important, and RABP is recommended to be over 85% [20, 21]. In our study, the rate of adequacy (81.2%) of the 3 L PEG was slightly lower than 85%, which may be related to the high BMI. A retrospective study reported that obesity was an independent predictor of inadequate bowel preparation at colonoscopy [5] , and each 1 kg/m^2^ increase in BMI increased the likelihood of an inadequate composite outcome score by 2.1%. However, whether increased BMI is predictive of suboptimal bowel preparation for colonoscopy is under controversial. In a prospective study on the by Fok [22] et al using a validated BBPS did not demonstrate an effect of obesity on bowel preparation using a low-volume bowel preparation. Moreover, a phase III, randomized, assessor-blinded, multicenter study found no significant differences among participants of all BMI groups receiving ready-to-drink sodium picosulfate, magnesium oxide, and citric acid oral solution [23].

Although, the 3L split-dose PEG regimen performed better than the 2 L PEG regimen, 81.2% of the rate of adequacy is not enough. Whether it is better to use a 4 L regimen or change the oral preparation than a 3L regimen is currently unknown. In one previous study [7], they found increased BMI is not predictive of inadequate bowel preparation for colonoscopy when receiving a split dose 4 L PEG solution before the colonoscopy. However, there are still lack of relevant study to compare 3 L PEG with 4 L or a higher dose in people with high BMI. A higher dose of PEG may lead to a better cleanliness, but at the same time it may cause side effects. We need to find a balance between the efficacy and tolerability of optimal dose of PEG for bowel preparation. Studies exploring the suitable intestinal cleansing method before colonoscopy is needed for the overweight and obese people.

The adenoma-detection rate (ADR), a quality indicator for colonoscopy, was recommended in the guidelines to be ≥ 25% [24]. The ADR is inversely correlated with the CRC [25, 26]. According to statistics, every 1% increase in ADR could reduce CRC incidence and mortality by 3% and 5%, respectively. Since the ADR is based on histological examinations, which limits its clinical application, the PDR can be used as a substitute. The study by Occhipinti [27] showed that the 4 L PEG scheme had a higher PDR (OR: 1.32, 95% CI: 1.07-1.63, *P*=0.011) and ADR (OR: 1.29, 95% CI: 1.02-1.63, *P*=0.038) than the 2 L PEG scheme. Similarly, our study revealed that the 3 L PEG regimen had a higher PDR than the 2 L PEG regimen (62.0% vs. 52.9%; *P*=0.015). Although CIR negatively correlated with the incidence of interval CRC [28], it is recommended to be ≥ 90%. In this study, the CIR in both groups was greater than 99%, which met the standard requirements. People receiving 3 L spit-dose PEG regimen were more likely to experience nausea (*P* = 0.001) than those with 2 L PEG, however the feeling was mild. Adjunctive drugs [29, 30], used during bowel preparation and chewing gum [31] may reduce adverse reactions.

Asians generally have a smaller build, and the BMI classifications are different in the east and west. According to the Chinese standard, individuals with a BMI (24-24.9kg/m^2^) were classified as overweight. However, those people were thought to be with a normal BMI in western country. In the subgroup analysis, 3 L split-dose PEG was superior to 2 L PEG for bowel cleansing in overweight individuals (BMI 25-29.9 kg/m^2^); however, this advantage was not significant in relatively normal (BMI 24-24.9 kg/m^2^) and obese people (BMI ≥ 30 kg/m^2^). Results from our study suggested 2 L PEG was not inferior to 3 L split-dose PEG in individuals with a BMI of 24-24.9kg/m^2^. As for obese people, 3 L PEG regimen may be not sufficient, which needs further exploration.

Constipation was identified as a predictor of colonoscopy preparation failure in previous research, and individuals with constipation might need more PEG [32]. Likewise, we found that patients with constipation were more likely to achieve adequate BP in 3 L PEG group than 2 L PEG group.

This study had several strengths. First, it was a multicenter RCT using the block randomization method. Second, the endoscopists were blinded to the bowel preparation regimen of the patients, which reduced subjective bias. However, there were still some drawbacks, for example, recall bias and the small sample size of the obese subgroup. In addition, 50 patients were lost to follow-up, we could not obtain information on adverse reactions. Moreover, there were no control subjects with normal BMI, and our subjects are not that overweight (especially BMI in the 30+ range). At last, split-dose regimen was not taken in the 2L PEG group. This prospective study can provide evidence for colonoscopic bowel preparation in relatively high-BMI individuals.

In conclusion, our multicenter randomized controlled trial study confirmed that 3 L split-dose PEG regimen was superior to 2 L PEG regimen in bowel cleansing before colonoscopy in people with BMI ≥ 24 kg/m^2^, especially with BMI ranged from 25 to 29.9 kg/m^2^, and those with constipation. Whether 4 L or a larger dosage is better for intestinal cleanliness is still a hanging matter. Further study is needed to answer this question in the future.

### Supplementary Information

**Additional file 1.**

**Additional file 2.**

## Data Availability

The data supporting the findings of this study are available from the corresponding author, Kai Deng, upon reasonable request.

## Abbreviations

BBPS: Boston Bowel Preparation Scale
BP: Bowel Preparation
BMI: Body mass index
CRC: colorectal cancer
CIR: cecal intubation rate
PDR: polyp detection rate
PEG: polyethylene glycol
RABP: Rate of adequate bowel preparation
RCT: randomized controlled trial
