# Supplementary material for: 3 L split-dose polyethylene glycol is superior to 2 L polyethylene glycol in colonoscopic bowel preparation in relatively high-BMI (≥ 24 kg/m2) individuals: a multicenter randomized controlled trial
Source: BMC Gastroenterol. 2023 Dec 5;23:427. doi: 10.1186/s12876-023-03068-9 (PMC10698874; doi:10.1186/s12876-023-03068-9)
Supplement: Supplementary file 1 — Additional file 1. [file 12876_2023_3068_MOESM1_ESM.pdf]

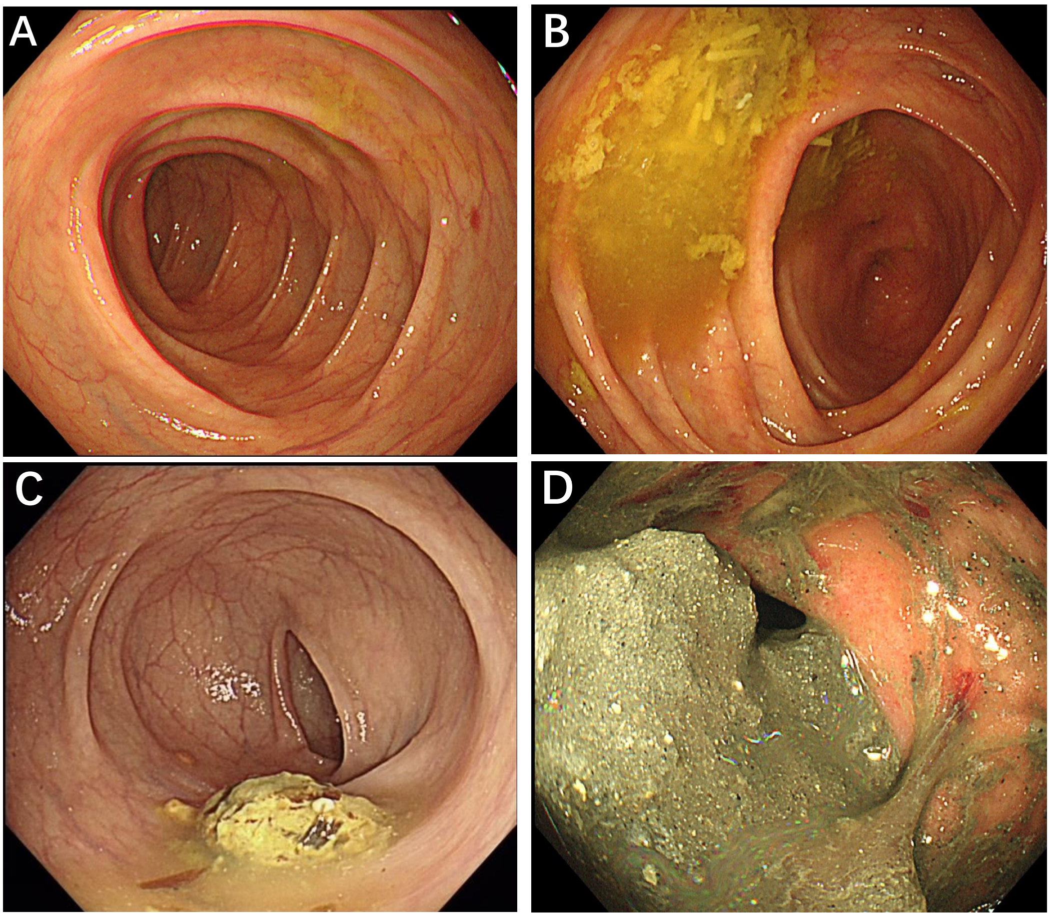

Supplementary Figure 1 Examples of original figures from different individuals on the cleansing level of colonoscopy by using the Boston Bowel Preparation Scale (BBPS)

A. Score 3: Entire mucosa of the transverse colon was seen well, with no residual staining, small fragments of stool, or opaque liquid.

B. Score 2: Minor amount of opaque liquid, but the mucosa of the right colon was seen well.

C. Score 1: Portion mucosa of the left colon was seen, but some areas were not well seen because of residual stool and opaque liquid

D. Score 0: Unprepared colonic segment with mucosa not seen because of solid stool that cannot be cleared.
