# Supplementary material for: 3 L split-dose polyethylene glycol is superior to 2 L polyethylene glycol in colonoscopic bowel preparation in relatively high-BMI (≥ 24 kg/m2) individuals: a multicenter randomized controlled trial
Source: BMC Gastroenterol. 2023 Dec 5;23:427. doi: 10.1186/s12876-023-03068-9 (PMC10698874; doi:10.1186/s12876-023-03068-9)
Supplement: Supplementary file 2 — Additional file 2. [file 12876_2023_3068_MOESM2_ESM.docx]

**Supplementary Table 1. The list of low-residue diets**

| food category | food choices available |
| --- | --- |
| staple food | porridge, white rice, soft noodle, bread made of fine flour, steamed cakes |
| meat | chicken, fish, rabbit meat, shrimp meat, all should be cooked until soft |
| eggs | steaming liquid egg, boiled eggs, egg soup |
| dairy products | fresh milk, pure milk, yogurt |
| beans | soft bean curd, tofu |
| vegetables | root vegetables, such as potato, carrot, winter melon, cucumber |
| fruits | fruit juice |
| cooking oil | vegetable oils，such as canola oil, olive oil, peanut oil |
